# Fate of petroleum-based and plant-based teabags exposed to environmental soil conditions for one year

**DOI:** 10.3389/fbioe.2022.966685

**Published:** 2022-09-06

**Authors:** Alicia Mateos–Cárdenas

**Affiliations:** ^1^ School of Biological, Earth and Environmental Sciences, University College Cork, Cork, Ireland; ^2^ Environmental Research Institute, Cork, Ireland

**Keywords:** teabag degradation, plastic pollution, bioplastics, soil, polylactic acid (PLA), cellulose, polypropylene (PP), environmental conditions

## Abstract

Petroleum-based plastics are materials which have provided important industrial benefits from being lightweight and having low production costs. However, plastic pollution is pervasive and ubiquitous on all environments. This has led some industries to rapidly introduce the so called ‘bioplastics’ into the market by switching the conventional ones for new plant-based alternatives with similar properties. However, little is known about the fate of such alternatives especially in the open environment. In this novel study, the degradation of teabags from eight different brands was investigated, five petroleum based (cellulose-PP blend) and three plant-based (cellulose, cellulose-PLA blend and PLA). The degradation was tested under real-environmental soil conditions over a 12-month period. Fourier Transform Infrared Spectroscopy (FTIR-ATR) and Scanning Electron Microscopy (SEM) techniques were used to examine the change in polymer makeup and surface degradation of teabags at 3 weeks, 3.5, 6 and 12 months. Teabag dry weight and any retrieved fragments were measured over time. Teabags that contained a plastic blended to cellulose were brittle or degraded into smaller fragments after 3 weeks in soil. Parallel to this, the cellulose layer also degraded in this short timeline. Petroleum-based teabags produced the highest numbers of PP fragments overtime and fragmented teabags were still found after 12 months. Plant-based teabags made of cellulose only or a blend of cellulose-PLA were absent from soil samples after 3.5 months, including no fragments. Contrary to this, teabags made of PLA which were marketed as completely biodegradable, persisted completely intact in soil throughout all time points. The novel results from this study provide a perspective on plastic degradation in terrestrial sources. Based on these findings, it can be recommended that teabags mostly made of cellulose or cellulose blended with a bioplastic present in a smaller ratio, are a better alternative to petroleum-based or pure PLA plastics, in terms of rapid environmental degradation. Further studies should focus on their ecotoxicity, additive presence, microbial degradation and life cycle in order to draw a full environmental assessment.

## Introduction

Plastic pollution has been widely monitored, especially in marine environments. It is understood that 80% of plastic pollution in the ocean originates from land and that coastal cities are a major source of pollution to the ocean (Jambeck et al., 2015; Critchell et al., 2019). From all the plastic ever made until 2015, only 9% had been recycled, 12% had been incinerated and the remaining 79% ended up in landfill or the environment (Geyer et al., 2017). With the raising concern of plastic pollution, some industries have rapidly switched their conventional petroleum-based products to plant-based bioplastic alternatives with similar properties. The terms ‘bioplastic’, ‘biobased’, ‘biodegradable’ and ‘compostable’ are often mistaken or used interchangeably. In general terms, a ‘bioplastic’ can be made in whole or in part of plant feedstock (ie biobased) but may or not be also biodegradable or compostable (SAPEA 2020). The most popular bioplastic types in the market are polyactic acid (PLA) and polyhydroxybutyrate (PHB), which account for the 60% of bioplastic production. Indeed, PLA was called the polymer of the 21st century (Balla et al., 2021). Currently, bioplastics are used in a variety of applications such as single-use products and packaging, textiles, electronics, biomedical equipment, construction, automotive (Ainali et al., 2022).

It is known that PLA can biodegrade in industrial compostable facilities, however their degradation in the open environment is still unclear. A study by Chamas et al., 2020 pointed out that the degradation rate of HDPE and PLA is very similar in the marine environment, but that PLA degrades 20 times faster on land. The biodegradability of bio-based plastics like PLA is not well understood under natural conditions. Little is known about the fate of plant-based blends in the environment and how do they compare to petroleum-based plastics. This study tested the long-term degradation of petroleum-based and plant-based teabags under environmental conditions.

The aims of the present study were 1) to visually compare any changes before and after tests using light and SEM microscopy, 2) to measure weight loss of teabags overtime as a proxy of degradation, 3) to analyse the effect of degradation on polymer composition and 4) to monitor the production of fragments overtime.

## Materials and methods

### Selection of teabag samples and preparation for tests

A range of tea brands that offer their products in teabags were carefully chosen based on popularity in the Irish market and in order to select a range of materials, covering both petroleum-based and plant-based polymers. Teabags from a total of eight different brands were purchased from three different local supermarkets in Cork City (Ireland) during November 2020. As shown below in Table 1, three out of the eight teabag types were made of plant-based materials and five were of petroleum-based materials.

**TABLE 1:** Selection of teabag samples from different brands widely available in Ireland at the time of purchase (November 2020).

Sample code	Teabag polymer material (November 2020)		
	Polymers	As shown in package	From brand’s website
Petroleum-based 1	Cellulose + PP	No mention	Contain plastic (PP) as sealant
Petroleum-based 2	Cellulose + PP	‘Teabags are compostable’	Contain plastic (PP) as sealant
Petroleum-based 3	Cellulose + PP	No mention	
Petroleum-based 4	Cellulose + PP	No mention	Contain plastic (PP) as sealant
Petroleum-based 5	Cellulose + PP	No mention	Contain plastic (PP) as sealant
Plant-based 1	Cellulose + PLA	‘Biodegradable plant-based pyramid^®^ teabags’	PLA Paper
Plant-based 2	Cellulose	‘Manila hemp bag stitched with organic string’	
Plant-based 3	PLA	‘Special biodegradable mesh pyramid bags’	Cornstarch

### Design of the outdoor experiment

The soil degradation experiments were set up under real environmental conditions in a south facing private garden in Cork City. The designation of a private location for this study is justified by the Covid-19 travel restrictions at the time of the start of the study and the restricted access to university facilities. In total, four soil degradation experiments were set up for a duration of 3 weeks, 3.5, 6 and 12 months. To investigate the process of degradation over time, individual teabags were buried in soil at approximately 20 cm depth using 36-hole seedling trays placed randomly in grass-free garden soil. Tea contents were removed from all teabags prior any tests by cutting a small hole in the top corner. A single empty teabag was placed in each hole (Supplementary Figure S1). Therefore, four replicates (four samples per teabag brand, one per hole) were run per tray and timepoint. As a control, brown paper bags and plastic bags were cut and buried individually. Potting compost substrate (Klasmann Peat Free Potting Substrate, Fuit Hill Farm) was used in this experiment to fill the holes and cover the testing trays. This substrate consists of 55% organic coir (coconut pith), 20% high quality compost from green waste and 25% composted wood fibre. Each tray was completely buried for 3 weeks (Tray 1), 2.5 months (Tray 2), 6 months (Tray 3), and 12 months (Tray 4) to allow real environmental degradation processes to take place (Supplementary Figure S2). Therefore, during this time (November 2020—November 2021), the samples were exposed to outdoor Irish weather conditions. According to data gathered by the Irish meteorological Service Met Éireann for Cork Airport (situated 8 km from the experiment location), the samples were exposed to an annual average of 110.97 ± 19.96 mm of total rainfall, an annual average of 9.89 ± 1.12°C temperature and an annual average 9.67 ± 1.27°C 10 cm soil temperature at 9:00 a.m. UTC.

### Teabag retrieval from soil and inspection in laboratory

After each test, the soil containing teabag samples was collected from each grid hole. Soil samples were stored in a cold room at 4°C degrees until further examination in the laboratory. A first visual inspection of each soil sample was undertaken to check for the presence of whole teabags or fragments. If found, each teabag or fragment were dipped in filtered water and subsequently cleaned with ethanol. Samples were separated and labelled in petri dishes and dried at room temperature for 24 h. All the soil samples were dried in incubators at 45°C degrees for 72 h. The dry soil was gently crashed with a mortar and pestle to break soil aggregates. After this, each soil sample was sieved at 4 and 1 mm. A second visual inspection was done on the two size fractions. An additional inspection under a stereomicroscope was undertaken on the smaller size fraction of 1 mm soil. All the fragments found on these further steps were separated and stored. Pilot density separation tests (Crichton et al., 2017; Courtene-Jones et al., 2020) were trialed but deemed unsuccessful for retrieving microplastics in this soil type by the author. Therefore, to avoid an underestimation of fragments released by teabags all the leftover dry soil was visually checked by the naked eye three times and a random fraction of 10 g of soil was examined under a stereomicroscope for smaller fragments.

In this particular study, the risk of contamination in the laboratory was minimal as the samples were of a known specific colour, shape and polymer. Nonetheless, general laboratory protocols for the work with microplastics were carried out especially when processing samples. All the sieves, trays and petri dishes were thoroughly cleaned with a sieve brush and paper between samples.

### Visualisation of samples using light and SEM microscopy

Light microscopy images were taken of teabags at ×8 and ×40 magnifications using a Leica EZ4W microscope. In parallel to this, small sections from each teabag were randomly selected, cut, mounted on 10 × 10 mm aluminum SEM stubs (Agar Scientific, UK) and gold sputter-coated for 30 s. All teabag samples were visualised at high vacuum mode, a working distance (WD) of 11 mm and at 35x, 45x, 65x and 150x using a Scanning electron microscope JEOL JSM-IT100.

### Teabag dry weight measurement

The dry weight of each teabag before tests was measured in mg using a Pioneer mass balance (Ohaus Corporation, USA). In order to compare different samples, the dry weight change of all the teabags was calculated as a percentage, being 100% each initial weight and following the below equation in which *tt* means test time (3 weeks, 3.5, 6 or 12 months), *W*_*tt*_ is the weight at any specific test time and *W*_*0*_ is the weight at t = 0 before any tests:

$$dry\;weight\;change_{tt}=\;\frac{W_{tt}\times 100}{W_{0}}$$

### Polymer characterisation of teabags and fragments using FTIR-ATR

Polymer types were confirmed using Attenuated Total Reflectance (ATR) Fourier Transform-Infrared (FTIR) spectroscopy. Unused empty teabag samples as well as any teabag material and fragments retrieved after each soil test were analysed using a universal diamond Perkin Elmer Frontier FTIR-ATR plate under the following parameters: 32 scans, range 4,000–650 cm^−1^ and a resolution of 4 cm^−1^. The polymeric composition results from all spectra were checked using Perkin Elmer’s Polymer Library as well as Open Specy community library (Cowger et al., 2021).

### Statistical analysis

Data were checked for normality using Shapiro-Wilk test. A Kruskal-Wallis with Bonferroni post hoc test were used. A difference was deemed significant where *p* ≤ 0.05. All statistical analyses and graphs were run using R software (version 4.1.3).

## Results

### Light and SEM microscopy images

**FIGURE 1:**
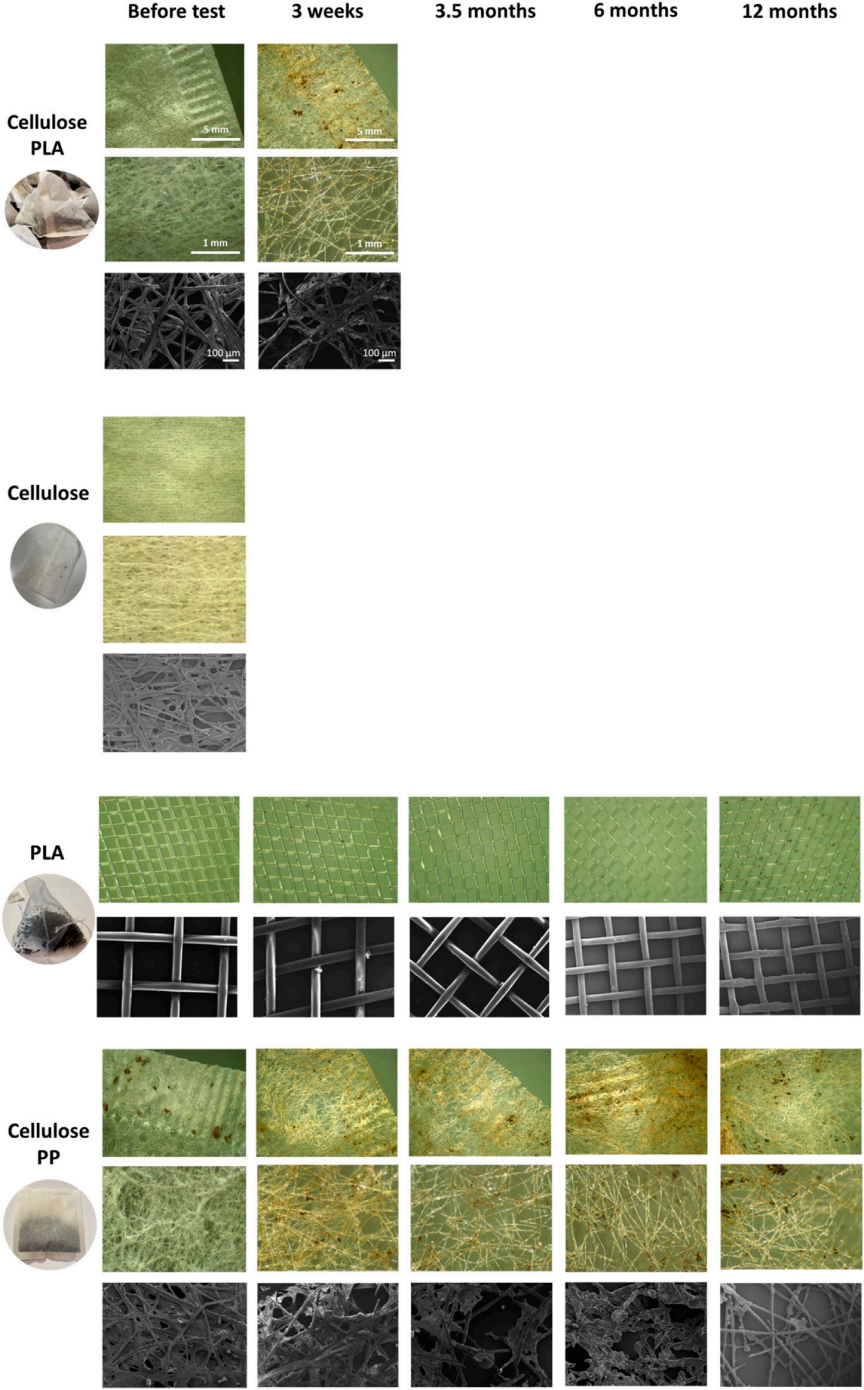
Light microscopy and SEM photos of the teabag polymer types tested in this study, PLA-cellulose blend, cellulose, PLA and PP-cellulose blend. Scale is shown in PLA-cellulose rows for all images.

In order to understand the degradation process, teabags were visually imaged using microscopy. Randomly selected teabags from each treatment were observed under light and scanning electron (SEM) microscopy before the test and after each time point. Cellulose-PLA blend and cellulose teabags were only imaged if they were present (Figure 1). A reduction in fibre density can be observed on cellulose-PLA and cellulose-PP blends before and after soil burial, whereas the PLA only teabags remained intact even after 12 months in soil.

### Dry weight change overtime

**FIGURE 2:**
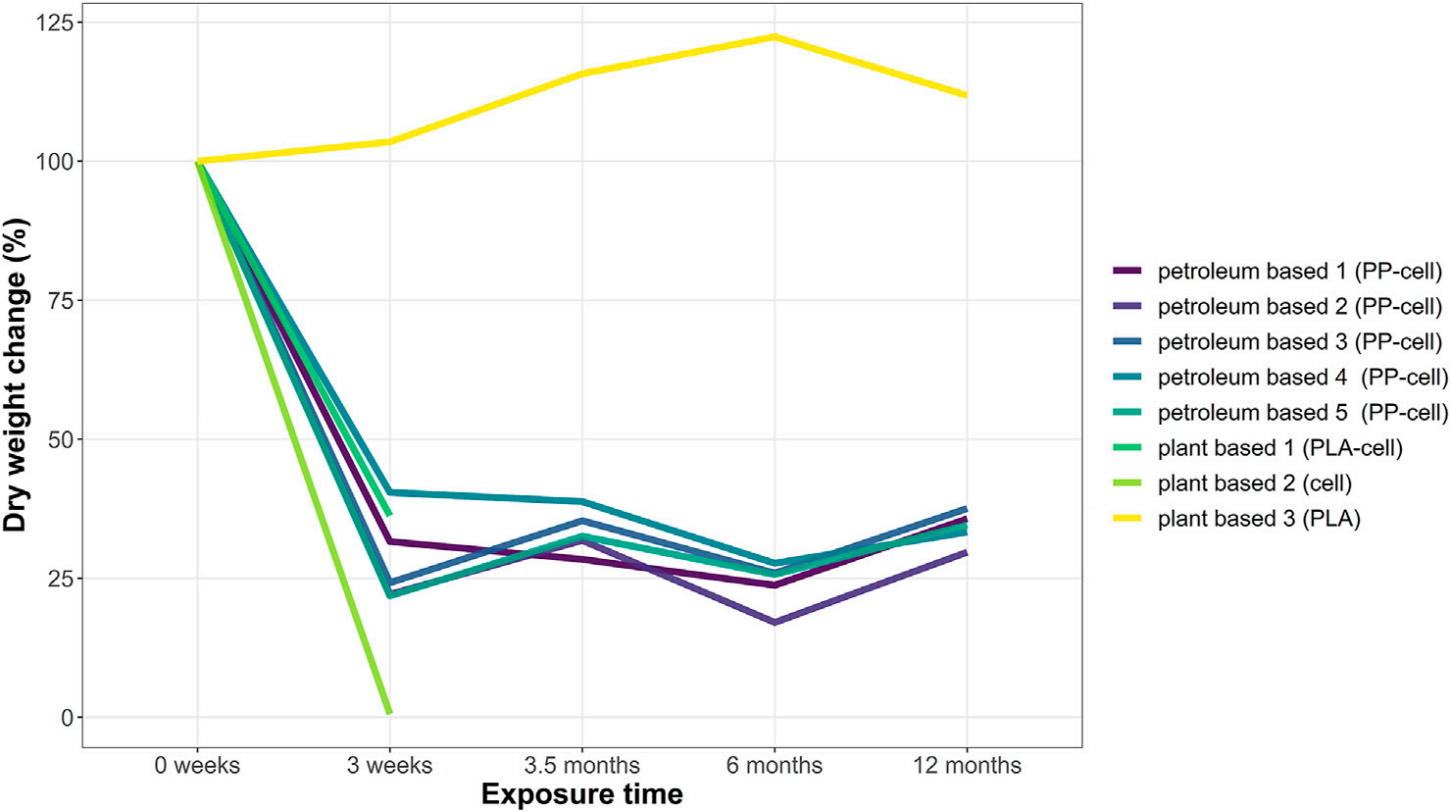
Dry weight change (%) of teabags over time. Four replicates were run per time point in each test grid. Each replicate consisted of one teabag buried individually. Data points show the total dry weight change (in percentage) of any remaining sample material per time point.

The before mentioned reduction in fibre density was also reflected in dry weight measurements. The dry weight of all teabags was measured before the experiments (t = 0 weeks) and at the end of each soil exposure (t = 3 weeks, t = 3.5 months, t = 6 months and t = 12 months). Overall, there was a significant decrease in dry weight over time on all samples tested except for plant based 3 teabag made of PLA, which increased slightly (Figure 2). The dry weight of all cellulose-based teabags dropped significantly at the beginning of the experiments (Kruskal Wallis, X = 11.69, df = 4, *p*-value < 0.05). A Bonferroni post hoc test confirmed that their weight significantly decreased at all time points compared to initial weight (t = 0). This is, for t = 3 weeks *p* < 0.05, for t = 16 weeks *p* < 0.05, for t = 6 months *p* < 0.01 and finally for t = 12 months *p* < 0.05.

### FTIR-ATR polymer composition of teabags before and after soil tests

The polymeric composition of teabags was checked before and after each soil burial test (Supplementary Figures S3–S6). The FTIR-ATR results from petroleum-based teabags confirmed the degradation of the cellulose layer after 3 weeks only (Table 2). This is, the main polymer detected before the soil burial tests from this teabag type was cellulose, however after degradation processes took place only PP was detected. Interestingly, FTIR-ATR analysis detected cellulose as the main polymer before and after the degradation tests for plant-based teabag 1. However, information shared privately by the brand to the author confirmed that these teabags also contain PLA as a sealant but in much lower ratio than cellulose.

**TABLE 2:** Polymeric composition of teabag samples before and after soil burial, as demonstrated by FTIR-ATR. It should be noted that plant-based 1 also contains PLA in lower ratio than cellulose (as shared privately by the brand) but was not detected spectroscopically.

Sample type	Soil test time				
	Before test	3 weeks	3.5 months	6 months	12 months
Petroleum-based 1	cellulose	PP	PP	PP	PP
Petroleum-based 2	cellulose	PP	PP	PP	PP
Petroleum-based 3	cellulose	PP	PP	PP	PP
Petroleum-based 4	cellulose	PP	PP	PP	PP
Petroleum-based 5	cellulose	PP	PP	PP	PP
Plant-based 1	cellulose^*^	cellulose^*^	No teabag material left in soil		
Plant-based 2	cellulose	—			
Plant-based 3	PLA	PLA	PLA	PLA	PLA

### Number and length of fragments

**FIGURE 3:**
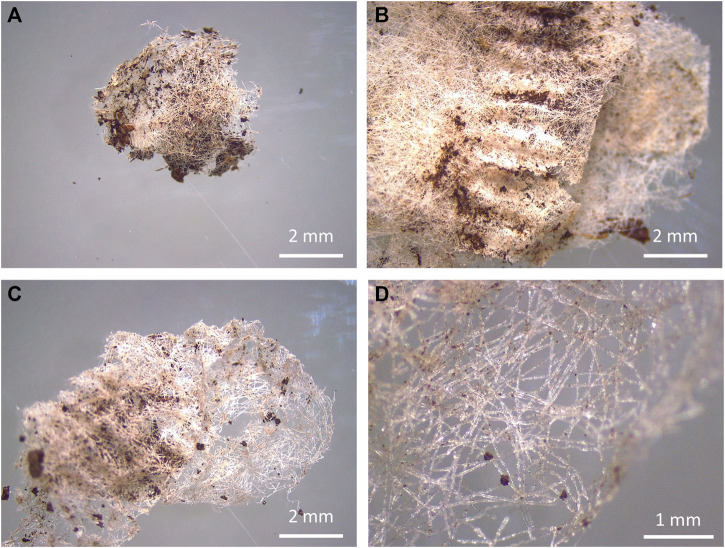
Teabag fragments (left) and degraded teabags (right) from teabags made of PLA-cellulose after 3 weeks in soil **(A,B)** and PP-cellulose after 3.5 months in soil **(C,D)**.

Fragments were the only shape type found in this study. Overall, a total of 136 fragments were retrieved among all soil tests and samples (Supplementary Table S1). From the total amount of fragments found, 83.82% belonged to petroleum-based teabags made of the blend PP-cellulose and the remaining 16.18% belonged to plant-based teabags made of the blend PLA-cellulose (Figure 3).

**FIGURE 4:**
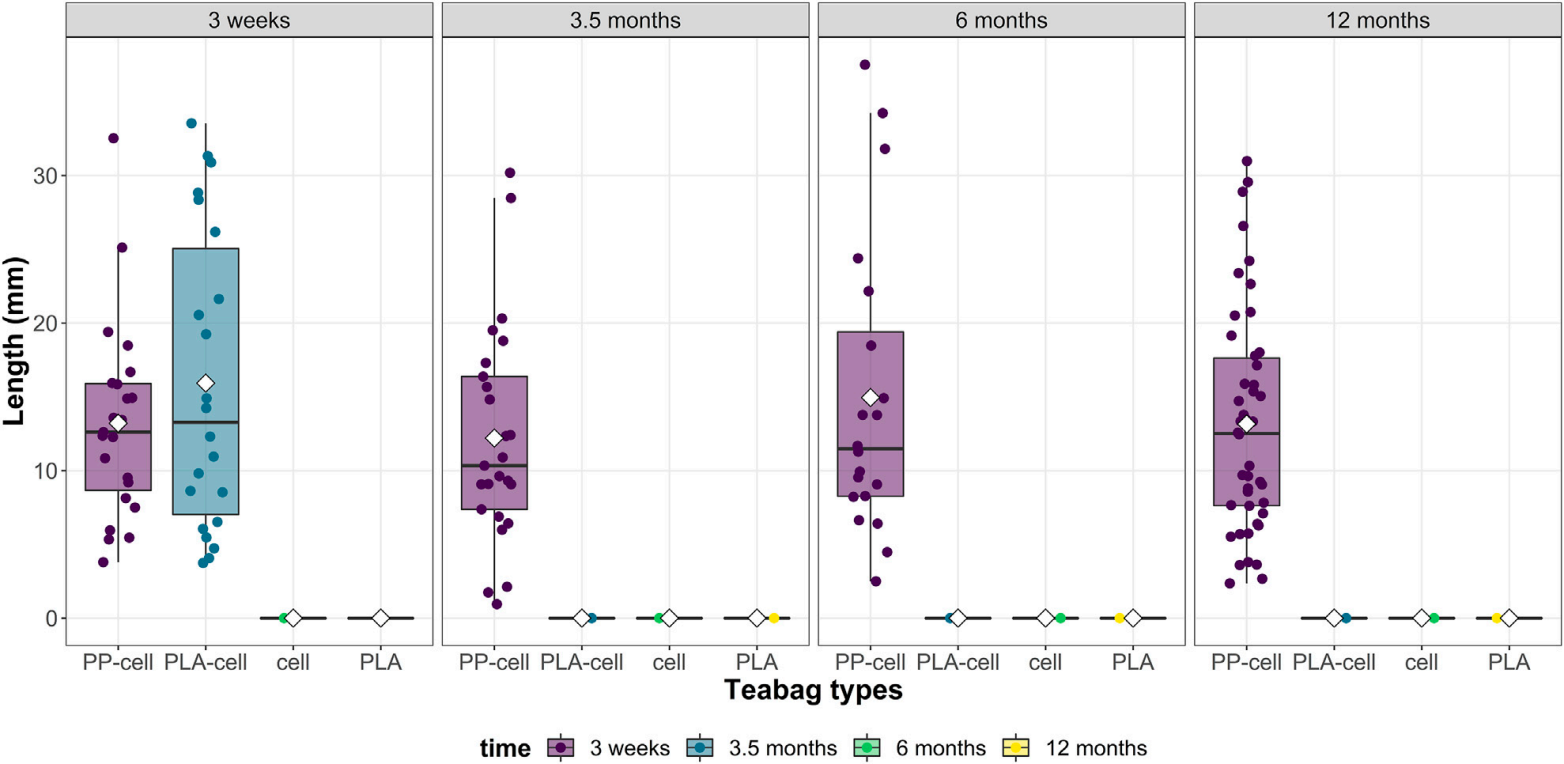
Total number of fragments in terms of size from the four teabag types across time points.

The total number of fragments produced was significantly different depending on teabag material (Kruskal Wallis, X = 9.78, df = 3, *p*-value < 0.05). Specifically, PP-cellulose teabags produced significantly more fragments than the other three teabag material types. The highest number of PP-cell fragments were found to be produced after 12 months in soil, which doubled the number of fragments produced by any other teabag material type, including PP-cell, at any other time point (Figure 4).

**FIGURE 5:**
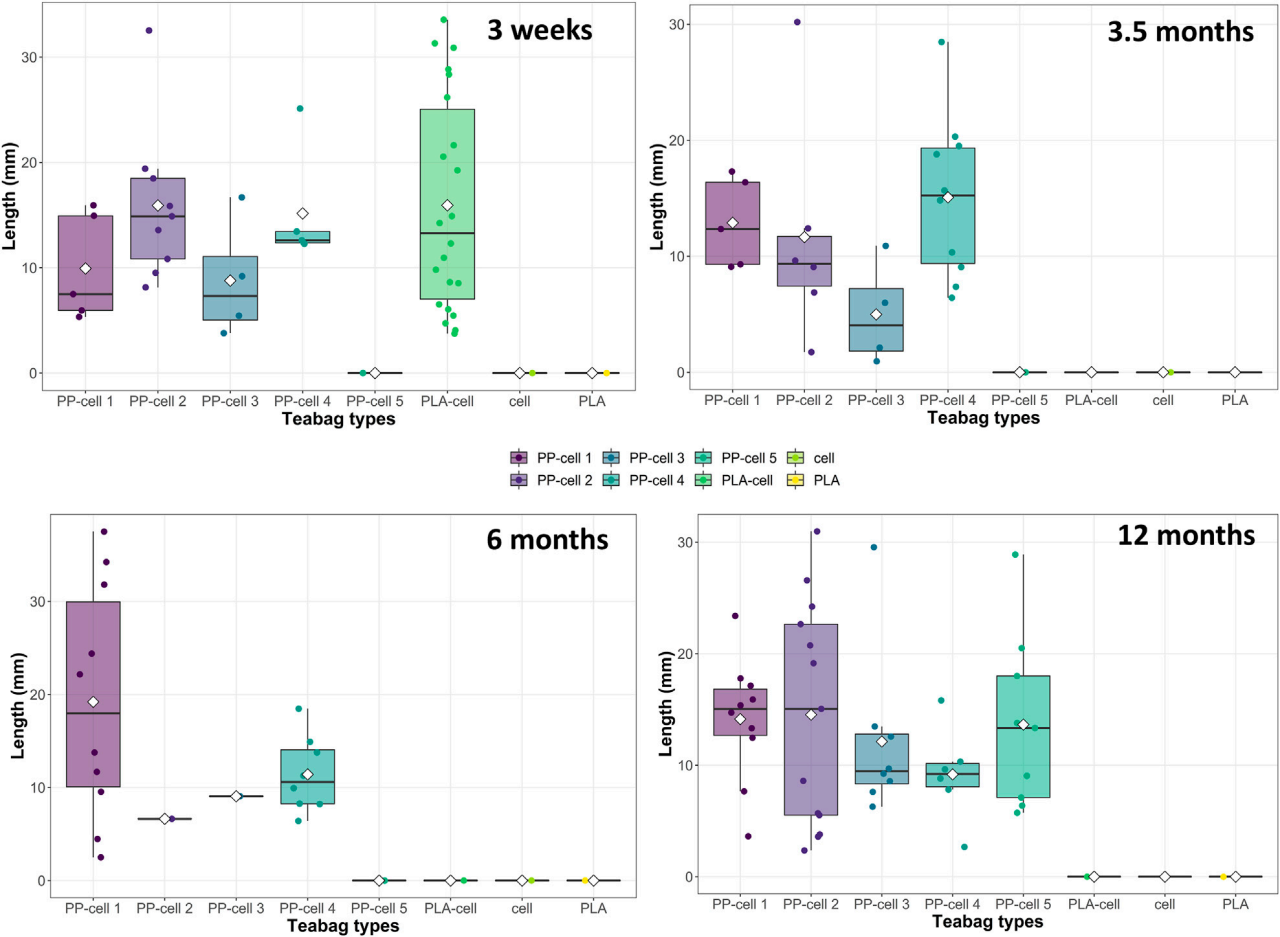
Total number and length of fragments produced by each teabag type over time.

Regarding fragment size, fragments were of various sizes depending on the teabag type and time exposure (Figure 5). From all the 136 fragments recorded, the average fragment size was 12.68 ± 0.70 mm length (mean ± SE) and the median was 12.32 mm. Overall, the average fragment sizes detected per teabag type were not significantly different overtime (Figure 5, Kruskal Wallis, X = 1.47, df = 3, *p*-value > 0.05). The smallest fragment detected in this study was of 949 µm in size and belonged to the petroleum-based teabag PP-cell 3 sample after 3.5 months soil exposure whereas the biggest fragment detected was of 37.51 mm length and belonged to petroleum-based teabag PP-cell 1 sample after 6 months.

Remarkably, plant-based 1 teabags (PLA-cellulose blend) and plant-based 2 (cellulose) were not detected in the soil after 3.5 months and 3 weeks, respectively, indicating that these teabags had apparently degraded. Contrary to this, all plant-based 3 (PLA) teabags remained completely intact in soil at all time points and did not produce any fragments. Lastly, regarding the positive controls, paper sheets had also degraded within 3.5 months of soil burial whereas the plastic sheets were still intact as one piece in soil after 12 months.

## Discussion

Here, the degradation of teabags was assessed for the first time. Degradability studies are commonly done under controlled conditions with ‘raw’ materials, however, there is a lack of studies that test the polymer degradation under environmental conditions and using consumer products. This study shows novel findings of a soil burial test over one year. Four different polymer types commonly found in teabags were tested, two of them were blends of PP-cellulose and PLA-cellulose and the other two were cellulose or PLA only.

A decrease in teabag density was visually observed on cellulose-plastic blends, both petroleum-based and plant-based, after 3 weeks in soil. This was also demonstrated by dry weight loss and decrease of microfiber density as shown in light microscopy and SEM images. After 3 weeks in soil, only PP spectra was detected in all petroleum-based teabags, demonstrating that the cellulosic layer of all PP-cellulose teabags had biodegraded. Therefore, the results demonstrate that cellulose fibres degrade faster than other materials, as shown before (Reese 1957). In fact, cellulose is commonly used as a positive reference material in degradability studies under controlled composting conditions (ISO 14855-1, 2012). The dry weight of PLA-cellulose dropped after 3 weeks in soil but their polymeric composition remained to be detected as cellulose. This could be explained by the variety in blends and variations in the polymer ratios produced industrially which can also directly impact the degradation process. For example, Narancic et al., 2018 generated several blends for testing such as PLA-PCL or PLA-PHB in ratios of 80/20. In their study, they showed that although some blends did biodegrade in soil and water, they did not pass standardised biodegradation tests such as ISO and ASTM. Also, Wei et al., 2015 showed that a PHB/PPW-FR blend consisting of potato peel waste fermentation residue could biodegrade faster than PHB alone. The specific blend ratios of the eight different teabags tested in this study are patented and therefore unknown. However, based on the FTIR-ATR spectra of teabags before and after the soil tests, it is suggested that cellulose is in a higher concentration than plastic by weight on all cellulose-based teabags, and hence directly impacting on their rapid degradation process in soil under low temperature typical from environmental conditions.

It was noted that PP-cellulose and PLA-cellulose were more brittle after the 3 weeks in soil and cellulose teabags had completely degraded. This could also be explained by the very significant drop in dry weight at the beginning of the soil degradation journey of each teabag, as natural cellulosic fibres are likely to degrade faster than anthropogenically manufactured polymers. Park et al. (2004) studied the biodegradability of cellulose fabrics in a soil burial test for 28 days. In this study, rayon and cotton had a higher biodegradability rate than acetate. In another study, regenerated cellulose films and a water-resistant film coated with Tung oil were found to degrade completely into CO_2_ and water after 2 months buried in soil. A 10–15% weight loss was reported after only 2 weeks in soil (Zhang et al., 1996). Remarkably, PLA teabags behaved completely differently from the other materials tested in this study. PLA teabags, in fact, remained intact in terms of weight and fibre density throughout the whole study. Furthermore, PLA teabags showed a slightly increased weight overtime, which could potentially be explained by a growing microbiota biofilm (MacLean et al., 2021).

Regarding the fragmentation of teabags in soil, no fragments (>900 µm) were found from cellulose and PLA-cellulose blend teabags after 3 weeks and 3.5 months, respectively. Contrary to this, PLA teabags remained completely intact over the whole duration of the tests and PP-cellulose teabags produced several fragments at all time points from 3 weeks up to 12 months. It should be mentioned that pilot density separation tests were attempted to find fragments or microfibers in the smaller ranges, however these tests were not successful. A limitation of the present study was on detecting fragments only bigger than 300 µm as this is the size limit that can be visually identified by the naked eye or under the stereomicroscope (Karlsson et al., 2020; Lv et al., 2021). Therefore, although the data suggest that the cellulose-PLA blend and cellulose teabags did degrade faster than the other two polymer types, complete mineralisation cannot be confirmed.

In the study presented here, the polymeric composition of the teabags appeared to influence their biodegradability behaviour. Polymer biodegradability can be explained by material properties such as crystallinity, degree of orientation, polymerization and hydrophobicity as well as environmental conditions and soil properties (Park et al., 2004). In general, different biodegradation mechanisms have been named to play a role in natural environments, these are moisture, temperature, pH, oxygen content or presence of microorganisms and fungus (Carson et al., 2013; Emadian et al., 2017; Kirstein et al., 2019). Biodegradation of plastics can be achieved under high temperatures and pressure in industrial facilities (Rudnik and Briassoulis, 2011). Biodegradation may be faster in soil and compost than in aquatic environments due to the presence of favourable biotic-abiotic conditions listed above. A burial soil test for 11 months found that material thickness influences the ability of PLA to degrade in soil (Rudnik and Briassoulis, 2011). As mentioned, crystallinity can negatively influence the degradation rate of (bio)plastics as higher crystallinity provides heat and chemically resistance to materials, contrary to amorphous forms (Vert et al., 1994; Karamanlioglu et al., 2017). Regarding microbial communities, microorganisms are able to better degrade organic materials present in biobased plastics such as cellulose or starch (Kale et al., 2015). Accinelli et al., 2019 showed that plant-growth promoting bacterium *Bacillus subtilis* promoted the degradation of a starch-based bioplastic from the average rate of 32–24 days in soil. Kumar et al., 2013 isolated LDPE degrading fungi from municipal solid waste and demonstrated the *in vitro* degradation of LDPE due to enzymes produced by *Aspergillus* spp. and *Fusarium* spp. Another study by Kathiresan 2003 showed a low degradation rate of plastic cups and grocery bags by *Pseudomonas* spp. and the fungal species *Aspergillus glaucus* present in mangrove soil. Microbial plastic degradation can vary depending on the chemical composition of the plastics. For example, no enzymes have yet been found to act on some high molecular weight polymers such as PP (Danso et al., 2019), which may explain the pervasiveness of PP fragments from teabags in soil overtime. Regarding different environments, bioplastic degradation may be faster in compost and soil than in aquatic environments and landfill (Folino et al., 2020). Cucina et al., 2021 found that PLA plastics from single-use cutlery and cups degraded slower than starch-based grocery bags. It should also be mentioned that PLA degradation in soil is much slower than in composting facilities, but faster than in aquatic environments (Karamanlioglu et al., 2017). This would indicate that longer times would be needed for the degradation of such polymers in marine sediments. Lastly, Napper and Thompson. 2019 showed that biodegradable, oxo-biodegradable, compostable, and conventional plastic grocery bags persist in different degradation stages when littered in sea, soil or open-air for up to 3 years.

The study of toxicity of different polymers was out of the scope of this study, however further studies should also focus on this matter in order to draw a full environmental assessment. It is known that microplastics may have different effects including mixed effects or no effects and nanoplastics pose a major risk than large microplastics. Very few studies have investigated the ecotoxicity of plastics from consumer products and the potential toxicity of bioplastics and its blends is still poorly understood. Zimmermann et al., 2019 investigated eight different polymers including PP from bottles, cups and packaging and PLA from cups, trays, bottles and lids. Baseline toxicity was strongly found on all PLA products whereas the results varied for PP and other petroleum-based plastics. Contrary to this on another study, PLA and PLA-starch blends had no effect on germination rate and plant growth of monocotyledon and dicotyledon plants (Rudeekit et al., 2012). Likewise, PLA and its blends did not have any cytotoxic, genotoxic and mutagenic negative effects on *Allium cepa* (Palsikowski et al., 2018). Further ecotoxicological studies are urgently needed to screen and compare the toxicity of both petroleum-based and plant-based plastics, including additive presence.

To conclude, the novel findings from this study showed how different teabag materials behave once in soil and under environmental conditions for up to 12 months. The results presented here indicate that pure cellulose or cellulose-PLA blend degrade relatively fast in soil, that cellulose-PP teabags produce plastic fragments that remain in soil after one year and that PLA teabags remain completely intact in soil after one year, despite being marketed as ‘biodegradable’. This highlights that industries need to better inform about the environmental degradation of their single use products in the market. Furthermore the rapid biodegradability of a material in the open environment should not be confused with active littering. Most importantly, regulations on bioplastic production and waste management should pressure producers to test the biodegradability of their materials under real environmental conditions and not only under standardised laboratory conditions. Also, to control this further, the legal definition of bioplastics should be more accurate and only include plant-based materials that are capable of biodegrade and to do so under environmental conditions. For this, more research is needed to understand the long-term (bio)plastic degradation under a range of environments.

## Data Availability

The raw data supporting the conclusions of this article will be made available by the authors, without undue reservation.
