# Supplementary material for: Fate of petroleum-based and plant-based teabags exposed to environmental soil conditions for one year
Source: Front Bioeng Biotechnol. 2022 Sep 6;10:966685. doi: 10.3389/fbioe.2022.966685 (PMC9485558; doi:10.3389/fbioe.2022.966685)
Supplement: Supplementary file 1 [file DataSheet1.docx]

Supplementary Material

**FATE OF PETROLEUM-BASED AND PLANT-BASED TEABAGS EXPOSED TO ENVIRONMENTAL SOIL CONDITIONS FOR ONE YEAR**

Alicia Mateos Cárdenas

**MATERIALS AND METHODS**

**Design of the outdoor experiment**

**
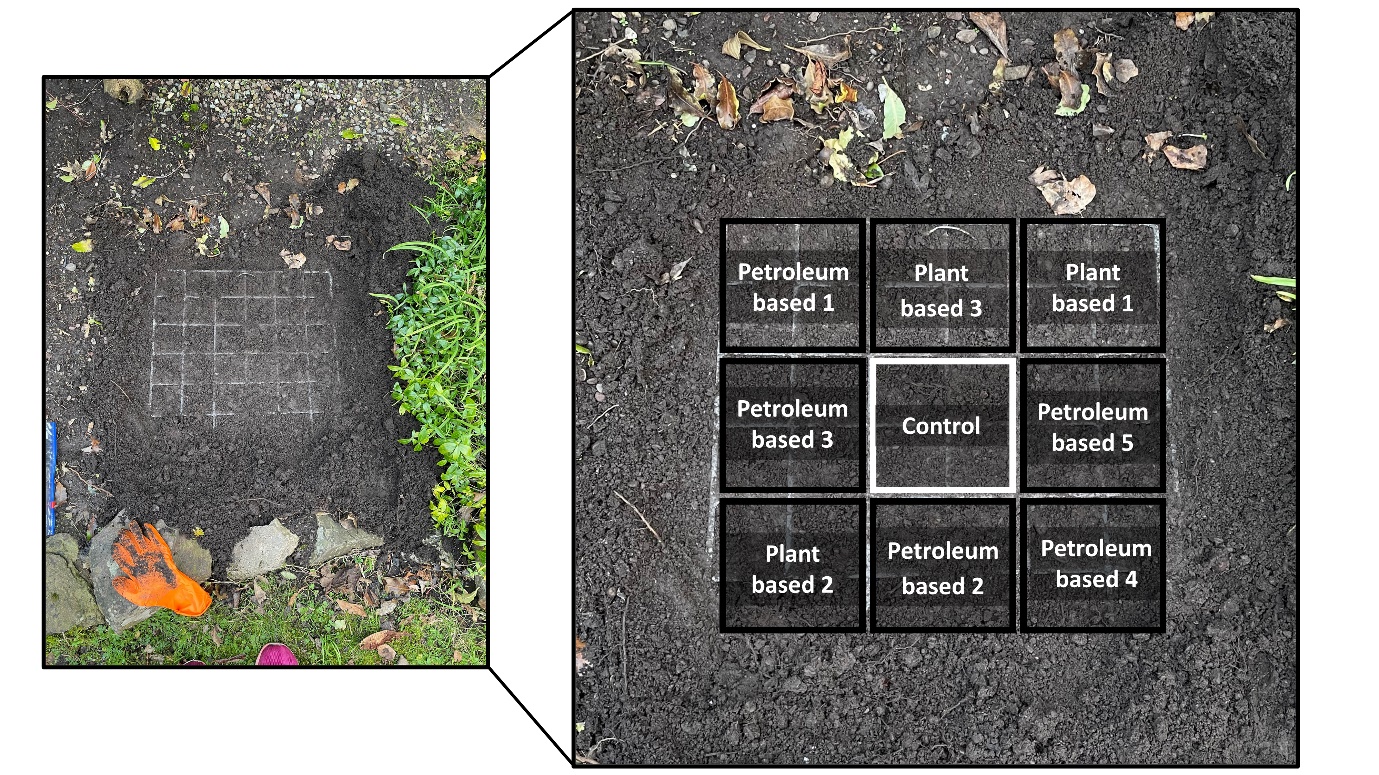
**

**Supplementary Figure S1.** Design of soil experiment using 36-hole seedling trays. One tray was set up for each time point. Each tray was divided into nine sections of four holes for each of the eight teabag samples plus a control consisting of a piece of brown paper and a PE grocery bag. A total of four replicates (N=4) were run by placing one empty teabag per hole within their randomly selected section of the grid. Picture on the left shows the main tray before being completely buried after being filled with the samples and remaining soil. Close-up image (right) shows a detailed explanation of the four replicates run per sample and time point.


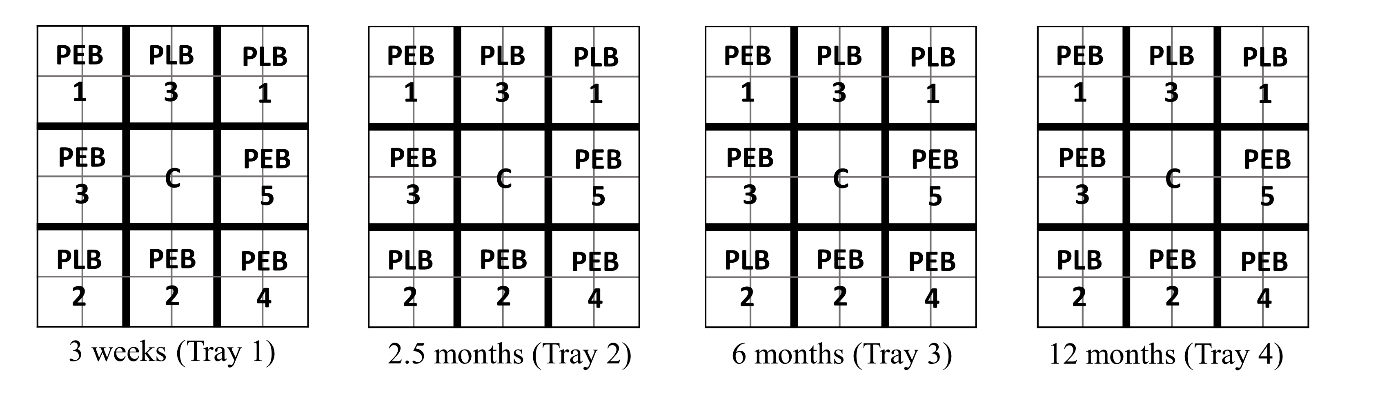


**Supplementary Figure S2.** Experimental design of the four time points. Eight brands were tested (N=4 replicates per teabag brand). Each teabag was placed in each hole. Hole groups were randomly allocated in the grids at the beginning of all experiments, being the five different Petroleum-based teabags PEB 1-5 and the three Plant-based teabags PLB 1-3.

**RESULTS**

**Supplementary Table 1.** Fragment sizes showed in mean ± SE per sample. The figure in brackets represents the total number of fragments. Blank spaces denote absence of fragments found.


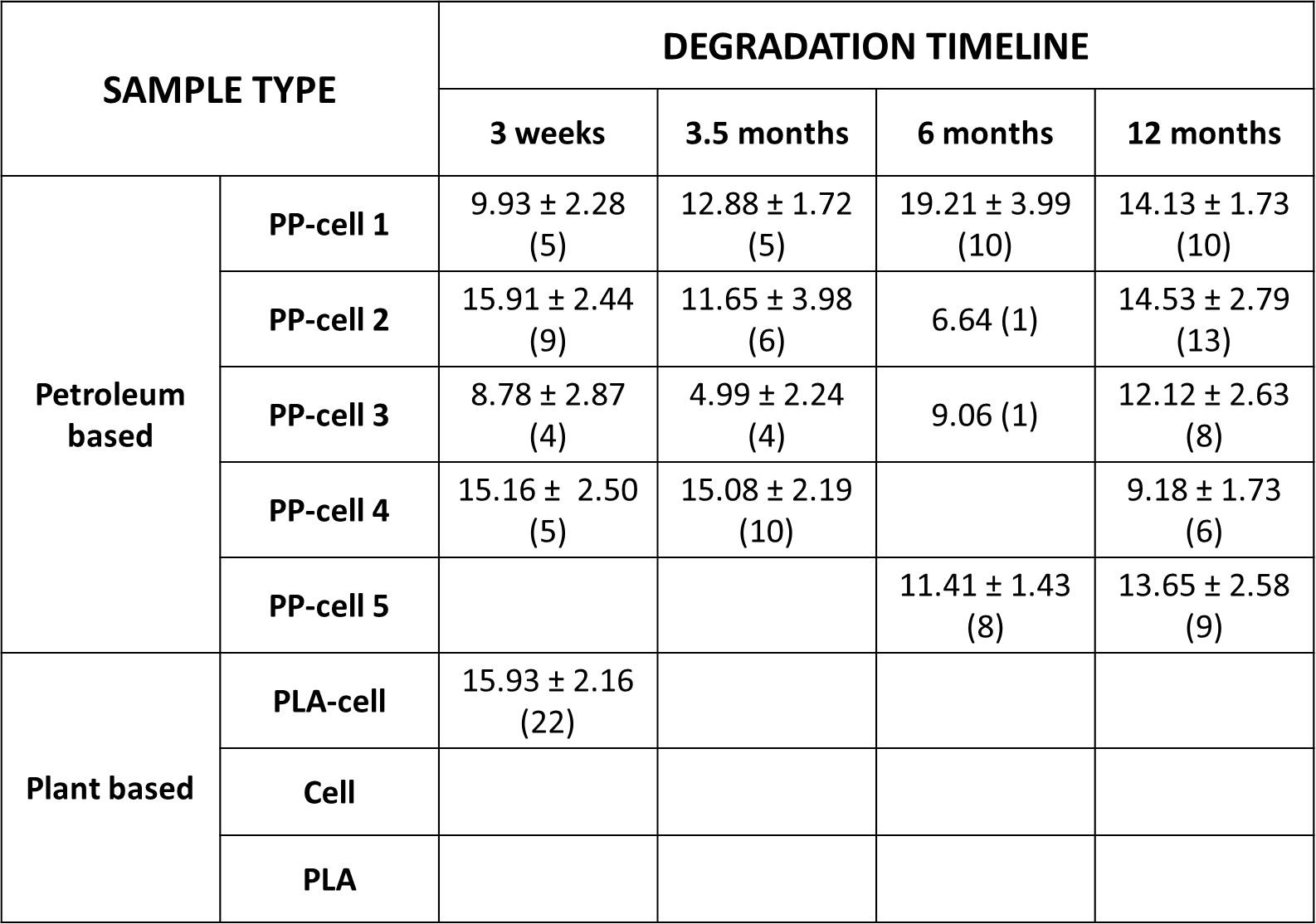


**
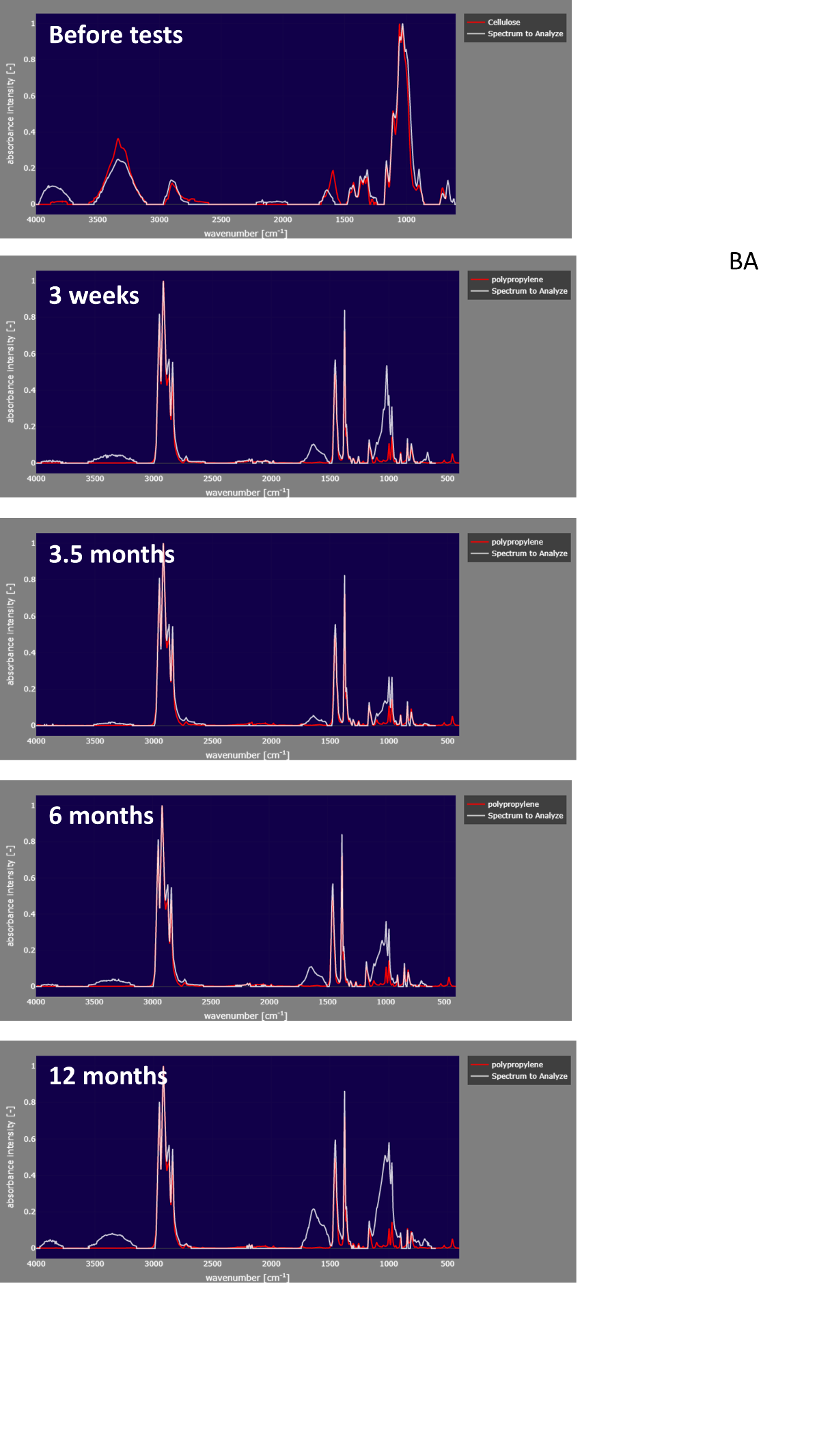
**

**Supplementary Figure S3.** FTIR-ATR spectra of petroleum-based teabag 1. Here petroleum-based teabag 1 is shown as a sample of all the five teabags in this study that are also made of the same blend PP-cellulose.


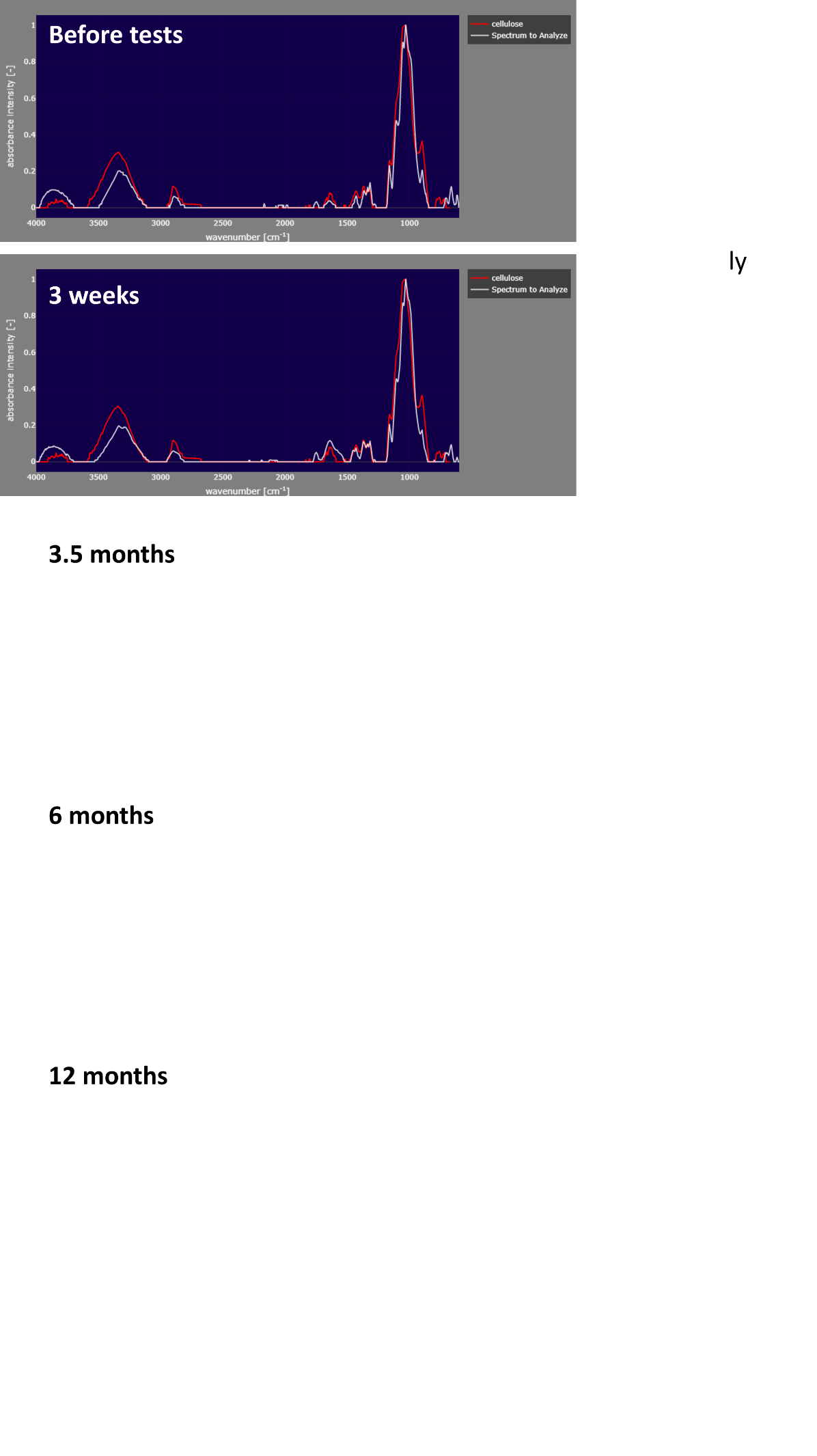


**Supplementary Figure S4.** FTIR-ATR spectra of plant-based teabag 1


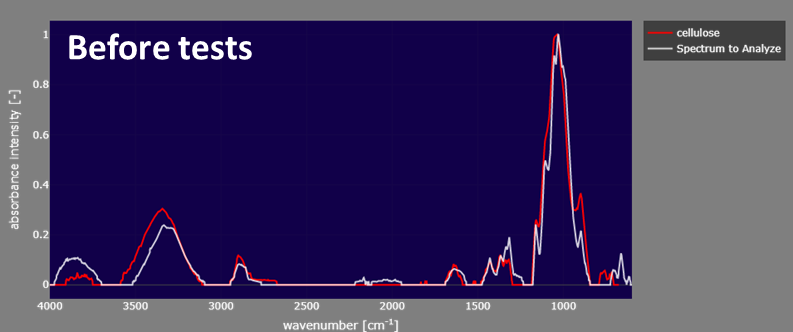


**Supplementary Figure S5.** FTIR-ATR spectra of plant-based teabag 2


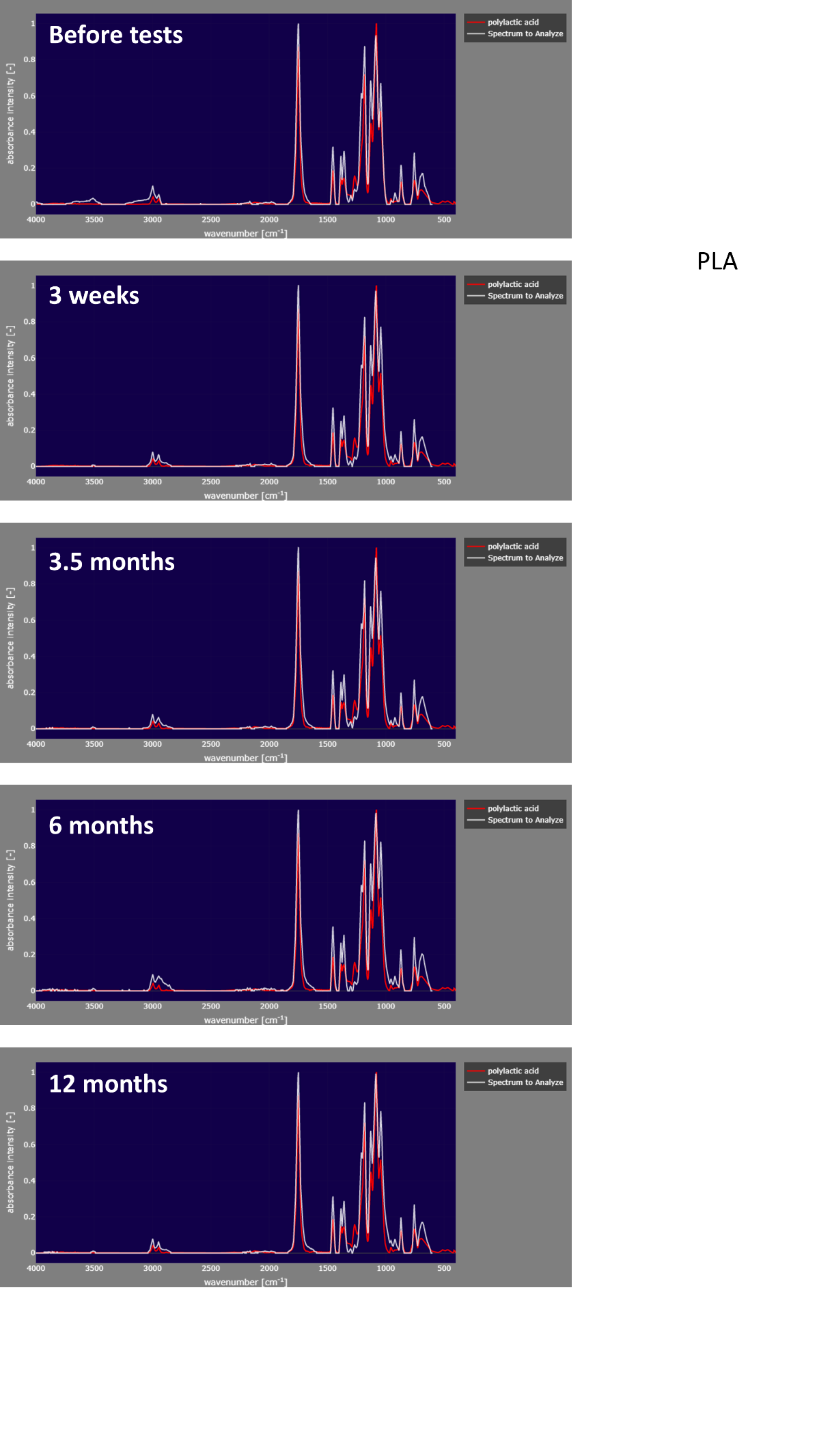


**Supplementary Figure S6.** FTIR-ATR spectra of plant-based teabag 3
